# Palliative Care in Advanced Dementia

**DOI:** 10.3389/fpsyt.2020.00699

**Published:** 2020-07-21

**Authors:** Yvonne Eisenmann, Heidrun Golla, Holger Schmidt, Raymond Voltz, Klaus Maria Perrar

**Affiliations:** ^1^ Department of Palliative Medicine, Faculty of Medicine and University Hospital, University of Cologne, Cologne, Germany; ^2^ Center for Integrated Oncology Aachen Bonn Cologne Duesseldorf (CIO ABCD), Faculty of Medicine and University Hospital, University of Cologne, Cologne, Germany; ^3^ Clinical Trials Center (ZKS), Faculty of Medicine and University Hospital, University of Cologne, Cologne, Germany; ^4^ Center for Health Services Research, Faculty of Medicine and University Hospital, University of Cologne, Cologne, Germany

**Keywords:** advanced dementia, palliative care, end of life, terminal symptoms, terminal care, advance care planning

## Abstract

Dementia syndrome is common and expected to increase significantly among older people and characterized by the loss of cognitive, psychological and physical functions. Palliative care is applicable for people with dementia, however they are less likely to have access to palliative care. This narrative review summarizes specifics of palliative care in advanced dementia. Most people with advanced dementia live and die in institutional care and they suffer a range of burdensome symptoms and complications. Shortly before dying people with advanced dementia suffer symptoms as pain, eating problems, breathlessness, neuropsychiatric symptoms, and complications as respiratory or urinary infections and frequently experience burdensome transitions. Pharmacological and nonpharmacological interventions may reduce symptom burden. Sensitive observation and appropriate assessment tools enable health professionals to assess symptoms and needs and to evaluate interventions. Due to lack of decisional capacity, proxy decision making is often necessary. Advanced care planning is an opportunity establishing values and preferences and is associated with comfort and decrease of burdensome interventions. Family carers are important for people with advanced dementia they also experience distress and are in need for support. Recommendations refer to early integration of palliative care, recognizing signs of approaching death, symptom assessment and management, advanced care planning, person-centered care, continuity of care, and collaboration of health care providers.

## Introduction

Living with dementia until the end of life is a growing challenge significantly impacting individuals with dementia, family carers and health care professional. Dementia has become ever more conspicuous given the increasing number of people affected by dementia worldwide, estimated to double every 20 years (1) and is one of the most common causes of death (2). In the advanced stage of dementia people are practically fully dependent on carers (2).

Recognized as a progressive, life limiting syndrome without curative treatment palliative care is applicable for dementia (3). The need for palliative care in dementia is expected to increase over the next decades (4). People with dementia have a similar symptom burden to people with malignant (5, 6) diseases, Palliative care focusing on quality of life for people with advanced dementia can improve symptom burden, prevent under treatment of symptoms and overtreatment with unnecessary and burdensome treatment and can also reduce caregiver burden and enhance caregiver quality of life (5, 7). However, they receive palliative care less often and experience high symptom burden (5, 8). Caring for people with advanced dementia poses different challenges than caring for people with mild to moderate dementia. Despite an increase in research there is less evidence of palliative care interventions (9).

This narrative review summarizes the current state of palliative care for people with advanced dementia. It illustrates specific prerequisites for palliative care in dementia, common symptoms, complications, and suitable interventions to relieve symptom burden, and concludes with recommendations for palliative care in advanced dementia.

## Special Prerequisites of Palliative Care in Advanced Dementia

Originally, palliative care emerged in the UK for cancer patients in response to insufficient care for the terminally ill. Eventually conditions other than cancer were acknowledged as being in need of palliative care. Independent of specific diagnosis palliative care should be provided for people with life-threatening disease and their families “through the prevention and relief of suffering by means of early identification and impeccable assessment and treatment of pain and other problems, physical, psychosocial and spiritual” to maintain or improve quality of life (3). Parallel to the development of palliative care dementia care developed separately; both based on the same values for enhancing care of people with dementia and improving quality of life (10). Notably lessons learned from palliative care for people with cancer cannot simply be transferred to palliative care for people with dementia.

The course of dementia is characterized by a prolonged decline in function with extensive limitations to cognitive, psychological and physical functioning in advanced stages (2, 11). With further progression, it becomes increasingly difficult to predict the course in the individual and in contrast to cancer diagnosis, it is difficult to predict when a person approaches advanced stage of dementia. Clinical care and research thus lack an agreed definition of advanced dementia, use different terms to describe the stage, and different measurements to classify dementia, e.g., the Clinical Dementia Rating Scale (CDR) (12) or the Global Deterioration Scale (GDS) (13), impacting comparability (14). Measurements exist to classify dementia stage, with variable use in different countries. Scales show different sensitivity of specific characteristics, e.g., restrictions in cognition do not necessarily correspond with restriction in mobility.

As dementia progresses people experience reduced or lost verbal communication abilities (2) with a consequent impact on care. Despite restrictions in verbal communication people with advanced dementia can use other means of nonverbal communication such as body tension or minimal movements, turning their head away, frequency of breath and paralinguistic signals are all means of communication to express their current wishes or needs (15–17). Agreement or rejection reactions in a situation can be observed, although interpretation of nonverbal communication varies between health professionals (15).

### Place of Care in the Last Phase of Life

In developing palliative care for people with advanced dementia, it is important to consider circumstances of care such as place of care or where the person dies. The majority of people with early stage dementia live at home with family members caring for them. Approximately one third of people with dementia are cared for in nursing homes (18, 19). A survey identified dementia in 68% of nursing home residents with only slightly more than half having recorded diagnosis and with more than half suffering from advanced dementia (20). People in the advanced stages of dementia spend most of their time in nursing homes (21). The likelihood of nursing home admission increases with age and severity of behavioural symptoms (19, 22–24) high burden of family carers is also associated with nursing home admission (24). People with migration background are less often cared for in nursing homes (25).

Although nursing homes are common places for end-of-life care (5, 20) and many people with dementia die in nursing homes or hospitals their preferred place to die is their original home (26–28). Nursing home facilities are the most frequent place of death in several high income countries (28–32) with a range from 93% in the Netherlands and 49% in Wales, for example. Hospital is the most common place of death in South Korea and France, while dying at the original home is most common in Mexico and Italy. Death in hospice facilities or palliative care units is very rare: pneumonia, renal failure and sepsis increase probability of dying in hospital (23, 28). Risk factors to for death during hospital stay were male gender, higher age, acute admission and Alzheimer’s disease (33). Dying at home or in a care home as preferred place of death was associated with higher functional impairment and treatment goal to relief symptoms only (34). In the United States, a recent analysis revealed a decrease in deaths of people with dementia in nursing homes and hospitals and an increase in the number of deaths at home or hospice facilities (35). Transitions of hospitalized nursing home residents with advanced dementia from hospital to hospice increased from 3% to 11% in the US (36).

### Dying With Dementia

It is assumed that approximately one third of those deceased over 65 years of age die with dementia as a main or additional diagnosis (37). An increase in mortality from Alzheimer’s disease has been found for Europe (38). Dementia is now the leading cause of death among women in England (39), and for Germany it was identified as the fifth most common cause of death. Ranked the sixth most common causes of death in the US, deaths from dementia doubled there from 2000 to 2017 (40). Still dementia may be under recognized as cause of death (2). Dementia is life-shortening (2, 41), people with dementia have a shorter survival than people of similar age with chronic diseases but without dementia. Duration of illness varies among individuals averaging duration from 3 to 6 years (37). Average post-diagnosis survival time for women (4.6–5.1 years) is longer than for men (4.1–4.3 years) (38, 39, 41), while life expectancy following diagnosis is determined individually by a number of predictors, e.g., age, sex, increased comorbidity, and type of dementia (42–44). People with vascular dementia or Lewy bodies have a shorter survival time than people with Alzheimer’s disease (45, 46).

As with disease with duration of disease, it is also difficult to predict duration of the different stages of dementia, as these vary strongly between individuals. Moderate and advanced stages represent the longest periods of the disease with an average of 12 months and women living longer in the advanced stage (47). People with Alzheimer’s disease over the age of 70 were found to spend on average 4 years in the stage of advanced dementia (21). In the UK, it is estimated that among people with late-onset dementia, over 12% have advanced dementia (39). Severity of dementia increases with age, as advanced dementia has been found in 6.2% of 65–69-years-olds, reaching 24.2% among those 95 and older (19). However, some people with dementia die before they reach the advanced stage of dementia (48). One quarter of people with dementia die with advanced dementia and half of them die with moderate dementia (49).

Common causes of death for people with dementia include pneumonia, cardiovascular disease, and sudden, unexplained deaths, cancer is mentioned less frequently (28, 50). In regional data, cardiac failure was the most frequent cause of death for 44% of cases, followed by pneumonia for 25%. About 13% died from malignant tumors in these people mostly died in hospices (28). Subdividing causes of death according to dementia forms cardiovascular causes were found more often in vascular dementias than in Alzheimer’s disease. Death from cachexia or dehydration occurred for half of people with advanced dementia and was more likely in case of a palliative care approach (51). When carers expected death, most of those persons with dementia died from dehydration or cachexia (52).

### Recognizing Approaching End of Life

People with advanced dementia suffer from a number of distressing symptoms (53). In their last 12 months, nursing homes residents with dementia suffer most common from restrictions in mobility, pain, and sleeping disorder. Further frequent symptoms at the end-of-life could be identified; problems with eating, trouble with breathing, apathy and anxiety. Contrary to this sleep disturbances, challenging behavior, agitation and depressive episodes occurred less frequently (54). Increases in distressing symptoms such as febrile infections and problems with eating and swallowing may be indicators for death in the next six months (5, 55, 56). Clinical complications such as respiratory infections are associated with highest symptom burden (55). Other results showed increased mortality coinciding with low body weight or low Body Mass Index (57). Prognostic assessment tools all include at least one criterion related to nutritional status, such as reduced appetite, inadequate food intake, malnutrition, or weight loss (58).

A retrospective study showed pain, fear, anxiety, agitation, and resistance to care as most frequent symptoms in the last month preceding dying and difficulty with swallowing and pain as most frequent symptoms in the final week of life (56). Septicemia and pneumonia were most common complications (17%) shortly prior death and indicators for death (53). Health professionals are required to critically reflect perception of symptoms and observations to align treatment and care according to the needs in the last phase of life (54).

Reliable estimating remaining lifetime and recognizing signs of an ending life can contribute to increasing the availability of palliative care in any care setting (58). Despite numerous efforts, it remains difficult to predict the end of life of people with dementia (53, 59, 60). Health professionals often overestimate remaining life time. E.g., (56), only half of expected deaths coincided with the actual time of death. At time of nursing home admission, staff expected only one percent of these nursing home residents with dementia to die in the next 6 months. Finally, 71% of the residents died during this period (61). There are indicators that make health professionals and family members aware of the approaching end of life and they respond to changes in people with dementia in the last phase of life. Thus, visits by relatives and conversations between relatives and caregivers showed an increase in the last seven days of life and almost all people with dementia died in the presence of a family member or a healthcare professional (54).

Physicians associated unexpected death with suffering and poor quality of life (62). Expecting death within the coming months showed positive effects on quality of end-of-life care. Prognosis estimation under six months was associated with fewer burdensome interventions in people with dementia (63). Among people with dementia explicitly expected to die a lower symptom burden was noticed and all of them received morphine (52).

Current instruments for estimating prognosis of lifetime remaining need to be improved in reliability. An assessment tool with good capability of predicting three-year survival after diagnosis (42) may be a good frame for planning palliative care in people with dementia. Thus, the criterion of FAST 7c is not very suitable for forecasting death. The Mini-Suffering State Examination Scale (MSSE) was tested as a 6-month forecast. A high MSSE value was associated with a short survival time and high mortality and may be used as a guide to estimate a 6-month survival time (64). In the event of a high MSSE score, a benefit from palliative care is expected. The development of Advanced Dementia Prognostic Tool (ADEPT), offered yet another instrument with moderate prognostic reliability in validation study (59).

To facilitate access to palliative care timely identification of people in need for palliative care is necessary. The Gold Standard Framework Prognostic Indicator Guidance can serve as an indicator starting with the Surprise Question “Would you be surprised if the person were to die in the next, months, weeks, days?” followed by general and disease specific indicators (65). Also, NECPAL tool includes the surprise question and presents a process of identifying and initiating palliative care process (66).

## Common Symptoms and Complications, Treatment Options

As the disease progresses people with dementia become more and more dependent on other persons for almost all daily activities, care and treatment, t suffering considerable limitations in the last year (11). As shown above people with advanced dementia suffer a range of complex needs and symptoms, (2, 5, 53–56) and symptom burden is similar to people with cancer or frailty (67). People with advanced dementia rarely express their needs and burdensome symptoms spontaneously, relying on their caregivers’ sensitive perception and interpretations of their verbal and nonverbal signs.

### Pain

Many people with advanced dementia experience pain; it is one of the most common symptoms and persists over the course of disease (53, 55, 56). Up to 63% of people with dementia suffer pain (6). Causes of pain are similar to older population and frailty, e.g., arthritis, constipation, and infections. Pain may influence other symptoms at the end of life, e.g., pain and depression were associated with each other, while people with lower levels of pain had lower depression scores (68). Behavioral and psychiatric symptoms are associated with pain (53). Symptoms, such as depression, agitation and challenging behavior can be indicators for present or not adequately treated pain as in people with dementia (69, 70).

Pain is a subjective perception, yet with dementia in contrast to cancer, patients self-reported pain is limited in dementia. Communication restrictions and restrictions in memory and executive function hinder the person in communication of their pain. In most cases, proxy rating is necessary based on observations by family carers and health professionals. However, missing experience and lacking knowledge of adequate tools may constrain recognizing pain. A lack of time and minimal interdisciplinary teamwork hinders pain detection (71). There are several instruments to measure pain in people with dementia. The Pain Assessment in Advanced Dementia (PAINAD) scale (72) is a frequently used tooland available in several languages. It evaluates behavioral categories breathing, vocalization, facial expression, body language, and consolability as indicators of existent pain. Person-tailored approaches, regular evaluation and adaptation of pain treatment contribute to successful pain management (73). Behavior is more correctly interpreted if the carer knows the person with dementia well and already over a longer period of time (74).

Advanced dementia is associated with more severe pain but still intensified pharmacological treatment mostly starts only at the end of life (75). People with primary dementia diagnosisreceive pain treatment less often than those with secondary diagnosis (29). In home care settings people with dementia were not even treated adequately with analgesics if they suffered already from pain previously (76). Moreover people with dementia experience pain even if treated against it (73). Adequate pain management improved symptoms of depression, apathy, and night time behavior (77).

Frequently used analgesics are paracetamol, metamizole, non-steroidal anti-inflammatory drugs, and opioids. These are associated with few side effects of paracetamol, e.g. liver injury (doses over 4gr/d) and inherent risks for non-steroidal anti-inflammatory drugs, e.g. kidney injury, gastrointestinal bleeding, allergic reactions or oedematogenic effects, and for metamizole: kidney injury, hypotension, or agranulocytosis. Non-pharmacological interventions may be used to relieve pain, e.g., massage, exercise, application of heat, or cooling packs. Relaxation techniques or giving the chance to rest and music therapy and presence of other persons may reduce pain (78). In addition to basic pain medication, movement-specific pain may be reduced by (additional) medication on demand before moving or daily care duties. Regular gentle movement or physiotherapy may help to maintain some mobility and reduce pain (74).

### Eating and Drinking/Eating Problems

Over the course of the disease difficulties in eating become more and more manifest with consequently reduced food intake and need for support with eating and drinking. People with advanced dementia have problems to swallow adequately. Moreover, they tend to keep food in their mouths, stop chewing or spit out food. In the last month of life difficulty with swallowing was found in 42% of persons and 32% exhibited observable weight loss (53). Advanced dementia is a risk factor for aspiration followed by pneumonia (79). Instances of reduced food intake dictate that acute medical events need to be examined, as possible causes for eating problems, these include. acute infections, pain, inadequate oral health, medication related side effects, and stroke (80). Dementia is often accompanied by deterioration in oral health and oral hygiene which, among other possibilities, may be induced by medication side effects of dry oral mucosa (81) and possible subsequent damage to the oral cavity and teeth. Living with sore mouth is very burdensome, causes pain, hinders use of dental prosthesis and often reduces food intake of people with advanced dementia. Educating caregivers about oral hygiene has great potential for improving the oral health of people with dementia (82–84).

Several options are available increasing comfort of food intake comfort for the person with dementia. Health professionals can assist with eating, they can offer preferred food (e.g., sweet) or finger food, and adapt the texture of food (74). To create pleasant environment a focus on the action of feeding and upright position may influence oral food intake positively and reduce risk of aspiration.

There is no evidence supporting benefits of tube feeding in overall health and survival (85). For percutaneous endoscopic gastrostomy (PEG) there were no improved short-term or long-term mortality or referral rates to hospital. Dementia at the time of procedure was a predictor for death in the following year (86). Insertion of PEG entails the inherent risks and burdens of the medical intervention. Assistance with eating and drinking is the preferred alternative. Complications with tube feeding are associated with additional burden of hospital admission (87). Family members should be supported in decisions on nutrition and they should be prepared for the situation that their loved one will experience problems with eating and drinking in the advanced stage of dementia (80).

### Infections With Fever/Pneumonia

Respiratory and urinary infections are very common in advanced dementia and as shown above can be a predictor of the end of life (5, 75). Often they are terminal events ending in death, and people with dementia have twice the risk dying from pneumonia compared to those without dementia (88). Further respiratory infections often result in burdensome symptoms such as breathing discomfort or transition to hospital (87).

Use of antibiotics for respiratory infections is very common and can improve survival of people with advanced dementia. For urinary infection there was no improved survival. Antibiotic treatment is accompanied with the burdensome treatment intervention of intravenous or intramuscular administration or hospital transfer and associated with reduced comfort for people with advanced dementia in their remaining lifetime (89). Different care goal for treatment need to be carefully considered, e.g., whether to treat a urinary infection with antibiotics or to provide optimum comfort for the person with advanced dementia. A high proportion of possible distressing antibiotic treatments available may be inappropriate in the light of prioritizing comfort as primary goal of care (80).

### Breathlessness

Breathlessness was found in 12%–52% of people with dementia (6) and can be a very distressing and frightening symptom as in other chronic diseases. Breathing difficulty often results from pneumonia and significantly reduces comfort of people with advanced dementia (90). Pneumonia with breathlessness is common at the end of life (5). Among nursing home residents with dementia breathlessness increased from 16%–26% to 52% in the last week of life (73, 89).

Breathlessness is frequently underreported and undertreated (91), it is important to increase awareness of breathlessness as an issue affecting people with advanced dementia (90). Due to restricted communication capacities, self-reporting of breathlessness, and observing the degree of mobility limitation due to breathlessness is rarely possible (MRC-breathlessness scale). To accommodate cognitive impairment near death the Respiratory Distress Observation Scale (RDOS) was developed, enabling observation of breathlessness in people otherwise unable to self-report (92).

If treatment options of the underlying cause of breathlessness are already optimized or too burdensome symptomatic treatment is required to relieve breathlessness. In hospice setting people with advanced dementia are more likely to receive symptomatic treatment for breathlessness, e.g., oxygen in case of oxygen deficiency, morphine, scopolamine, or hyoscyamine than in a nursing home setting. Morphine is the only pharmacological treatment with evidence relieving chronic breathlessness in the advanced diseases, but only few people receive morphine (92). As nonpharmacological interventions, it may reduce breathlessness to help the person to adopt bodily positions to make breathing easier, e.g., upright sitting with arms supported at side or forward lean position. The sensation of fresh air to cool the facial area of nose, cheeks, and mouth by open windows, a fan, or cool, damp wipe may reduce perception of breathlessness (93). To keep mouth moist may also reduce breathlessness and enhance quality of care at the end of life.

### Neuropsychiatric Symptoms

Agitation and apathy, depressive and behavioral symptoms are common symptoms in the course of the disease (94). Agitation was recognized to be the most common and persistent symptom in 57%–71% of people with dementia decreasing in the last week of life (75). Neuropsychiatric symptoms were associated with lower quality of life and unmet needs as pain and social needs were associated with verbally agitated behavioral symptoms (95).

There are instruments to assess neuropsychiatric symptoms in people with dementia as proxy rating. The neuropsychiatric inventory (NPI) (96) is common and scores twelve behavioral symptoms, e.g., hallucinations, anxiety, agitation, and apathy with a high score as indicator for high symptom burden.

In view of aggregating risk factors of multimorbidity at high age people with dementia are at high risk to suffer from delirium. Although reversible delirium has negative effects on the course of the disease, survival time and care dependency (97). Behavioral changes due to delirium can arise suddenly whereas they develop gradually in dementia. Recognizing symptoms and treating possible causes, e.g., pain or infections are basic to manage symptom burden. There is a lack of pain assessment in people with agitation (98).

Adequate pharmacological and non-pharmacological pain management is essential to diminish physical causes of neuropsychiatric symptoms. People with advanced dementia have rare contact to specialist health care as psychiatrist for symptom management (53). There is controversy about possible side effects and effectiveness of antipsychotics, e.g., haloperidol or risperidone in palliative care patients. Nonpharmacological strategies may be more beneficial than risperidone or haloperidol (98).

Non-pharmacological interventions tailored to the individual may be more appropriate to treat behavioral symptoms (99), e.g., aromatherapy, massages of hand, or foot can be effective. Presence of a carer and being in interaction with others and individualized interventions may help to reduce symptoms like agitation, aggression, apathy, anxiety (72, 100). As a result of the discussions initiated by the publication of Agar and colleagues (98), a differentiated delirium treatment is recommended (101, 102). The combination of non-pharmacological and pharmacological approaches is required as well as a targeted treatment of individual symptoms of delirium. Sedatives or hypnotics (e.g., zopiclone, midazolam) are recommended for sleep disturbance, sedatives (e.g., midazolam, promethazine) for agitation or psychomotoric disorder, and antipsychotics (e.g., haloperidol, quetiapine, olanzapine, aripiprazole) for psychotic symptoms, but in low ()! doses.

## Special Issues of Palliative Care in Dementia

### Decision Making and Advance Care Planning in Advanced Dementia

In the course of dementia it is frequently necessary to make treatment decisions. In contrast to cancer patients who usually can take part in decision-making, restricted capacity of communication and cognitive function implies special impedes as decision making is typically based on informed consent. Due to temporal or permanent lack of decisional capacity surrogate decision making may become necessary, e.g., by family members and health care professionals (103, 104). Still shared decision making with the person with dementia, family caregiver and health professionals is the goal (105). People with dementia may verbalize their treatment wishes and needs in the early stages of the disease and may prepare written advanced care plans. Decisional capacity is prerequisite for autonomous decisions and at best starts before restrictions limit patient’s capacity. But already in early stages capacity to follow conversation, to make informed decisions can be limited (103). Other means of communication and therefore the ability to be involved in decision making and everyday matters may be preserved (15, 16).

Advanced care planning (ACP) is mentioned frequently as one key factor for good end-of-life care (17, 80, 105, 106). It aims at implementing patient’s individual values and preferences regarding possible therapeutic decisions for future health conditions. Persons concerned express their wishes regarding care preferences of treatment, caregiving, psychosocial and spiritual aspects. It is a communicative process confronting the person with dementia, family carers and health professionals with the possible course of dementia, death, and dying (2, 5) with the chance to strengthen communication of all stakeholders. Discussing values and preferences may highlight discrepancies between families’ and patients perception on treatment preferences (107). There is disagreement about optimal time for initiating ACP in the course of the disease, e.g., to start early after diagnosis (103, 105), and who should be responsible for. (108). In the advanced stage of dementia often proxy decision making is necessary and surrogates can be challenged to make existential decisions on behalf of the person with dementia. Family carers often feel not prepared to make decisions in case of deterioration or approaching death (107) and preferences expressed prior might contradict current behavior. Surrogates and health professionals implement patient’s preferences and values involving the person with dementia as much as possible tailored to current capacity (104). Most family carers choose maximizing comfort as primary goal of care (109). Without ACP people with dementia are at risk to receive burdensome interventions at the end of life (5, 110). Despite provision of palliative care elements transition of care goal to comforts mostly happens shortly before death (96, 106). Research showed an association of written ACP and quality of care in the dying phase. Emotional distress at the end of life such as fear and anxiety were less distinctive in case of written ACP. No effect was found in oral communication only between carers and people with dementia (111). ACP was associated with less tube feeding (85) and contributed to minimize hospital admissions (110) and increased the use of hospice structures (112). In comparison to people with other advanced diseases in hospital care people with dementia were less likely to receive intervention opposed to their treatment goal of comfort (113).

Still only some people with dementia have a written advance care plan (53, 107) whereas financial matters and questions about the person providing legal support are usually recorded in writing. Possible reasons for this include a lack of knowledge and awareness of the possibility of ACP or lack of support when preparing the document. Timing to initiate ACP is difficult or informal arrangements appeared to be more pleasant. For people with dementia and their relatives, ACP can go along with great uncertainty and emotionally stressful conversations (109, 114). People with dementia or their family members might not feel comfortable to discuss future situation of deterioration. Further obstacles are lack of training for health professionals to initiate ACP, lack of clarity on standards for documentation, how to access document when required and lack of appropriate funding (103). The ACP should be reviewed regularly and in case of change in health condition (105, 107). Various strategies to improve ACP were initiated, e.g., education of family carers about the course of the disease, detailed conversation about the end of life jointed with documentation of preferences, enough time and atmosphere for sensitive discussion of issues (107). ACP process with an educational component can provide more security for family carers in their decision-making processes. Family carers may be enabled to make decisions at the end of life together with the attending physicians (108). A resource-oriented view on dementia instead of a deficit focus can influence ACP regarding life-prolonging treatments of acute events with high chance of recovery (115).

### Family Carers

The majority of people with dementia are cared for by their relatives at their home. In most cases, main family cares are women: spouses, daughters, and daughters-in-law (18). Caring for a close relative with dementia entails various burdens and restrictions for family carers. They suffer from psychological and physical restraints and have a reduced quality of life (116, 117), they are in need of information and support during the course of the disease and the various phases of the grieving process (118, 119). Family carers and professional carers estimate the burden on caring relatives as high. Carers of people with Alzheimer’s disease appear to be more burdened than carers of people with vascular dementia (120). If family carers are seriously strained or are unable to provide care, the likelihood of admission to hospital or nursing home increases (24, 121). Some family carers experience admission to long-term care facility as burdensome or regard it as their failure (122). Evidence for supportive intervention in the process is still required (123).

Family members of people with advanced dementia living in nursing homes also experience distress. They reported psychological stress such as depressive symptoms (26%) and anxiety (41%). Key concerns of the relatives were the relationship with caregivers, understanding the course of the disease and emotional reactions to advanced dementia. They appreciate to have the possibility to influence care at the end of life. A feeling of loss of control often occurred when moving into a nursing home (122). If available, close family members as intimate can provide essential biographical information to get to know and understand the person with dementia or provide impulses for person-centered care to meet the individual’s needs (74).

Only around a fifth of family carers felt informed about what to expect at the end of life and usually initiated discussion (124). They are little accompanied in their emotional burdens and are at risk of prolonged grief. Grief and feeling of loss do not necessarily begin with the death of the affected person, but already earlier in phases of the personality change and when the relatives are no longer recognized (118, 119, 122).

Various instruments are available for assessing the burden of family caregivers, most of which have been developed for a home care setting. The Zarit-Burden Interview is widely used internationally in research and practice. The instrument comprises 22 items on areas of mental and physical health, financial situation and the relationship with the person with dementia (125). It is available and validated in several languages and as short form. For use in long-term care facilities the Caregiver Reaction Assessment Scale (CRA) (126) is available, also validated for various languages. These instruments aim to target outpatient care and cover all stages of dementia. There are some shortcomings of these instruments used for specifically recording the burden on relatives of nursing home residents with advanced dementia (22).

### Barriers for Good Palliative Care for People With Dementia

Despite increasing knowledge of optimal care and the recommendation for early integration of palliative care in the course of disease, accompanied by disease modifying treatment (12), a number of reasons for barriers of good palliative care for people with dementia were identified (119, 127).

Diagnosing dementia in the early phases can be challenging and lengthy, leading to delays early access to palliative care (118). Dementia is often not acknowledged as terminal and life limiting disease and consequently end of life is not taken into consideration or adequately addressed (14). Another barrier is the difficulty in predicting the duration of the disease or its anticipated course in contrast to cancer. Although experts have determined the average length of the different stages and length of the disease, accurate individual predictions remain challenging. Notably, people with dementia have limited access to hospice and palliative care (118, 127).

The symptom burden of people with dementia is similar to that of oncological diseases, but their course has a prolonged decline (11). It is challenging to reliably identify and manage symptoms reliable (127). The communication restrictions encumbering of people with advanced dementia (2) make it difficult to reliably recognize distressing symptoms such as pain or shortness of breath or anxiety. Additionally, instruments for assessment in clinical practice are lacking, compounded by the marginal usage ACP for the end of life which would enable taking sufficient account of the wishes and needs of the person with dementia (5). Diminished patient capacity to communicate wishes and needs and to make decisions for treatment preferences challenges health professionals and family carers in attempts at providing optimal care. Some health professionals lack knowledge about dementia and experience in the care for people with dementia at the end of life (107). Palliative care teams have expertise in providing symptom relief and end-of-life care but may not have the ability to deal with communication difficulties and behavioral symptoms of people with dementia (128). Family carers often experience a high burden of caregiving and receive too little support over the entire course of the disease to provide home care for their relative with dementia (118).

Another limitation contributing tosub-optimal palliative care in dementia is the lack of sufficient time to care for the individual affected and insufficient staff resources (127, 129, 130), demonstrated for nursing homes in general. General practitioners’ overall lack of time prevents home visits necessary for palliative care (129). Optimal care at the end of life demands that, systemic prerequisites must be met, such as ensuring sufficient funding (119). National framework and regulatory prerequisites for providing palliative care for people with dementia are necessary. Many countries have national dementia strategies, not all of them include recommendations for optimal palliative care in dementia or issues are named differently (131).

Regardless of all efforts there is still no consensus on palliative care in dementia (127). The applicability and appropriateness of palliative care for people with dementia was also one of the controversies in the development of the EAPC White Paper on optimal palliative care for dementia (132). There is a great need for robust study results on complex interventions at the end of life (9). The optimal timing to integrate palliative care or adapt care goals or simultaneous goals to modify disease and to provide comfort is still in discussion (17).

### Complex Interventions in Advanced Dementia

As people with advanced dementia suffer complex symptoms and still have numerous and complex physical, psychosocial and spiritual needs they are in need of multidisciplinary health care. By comparison with early stages of the disease there is less knowledge about needs in advanced dementia (133). Physical needs are related to adequate symptom relief and basic care needs. It is complex to meet the numerous and differentiated psychosocial needs comparable to people in moderate state of dementia, e.g., need of enhancing personhood, communicating and being in contact with others, participating in everyday life or feeling save and familiar (74). Unmet social needs for activity contributed to discomfort and behavioral symptoms (95). Needs being important in mild or moderate stage of the disease, e.g., financial needs and cognitive strategies for coping with disease (134, 135) are less direct needs at least for the people with advanced dementia themselves.

To address requirements of people with advanced dementia and their family collaboration of psychiatry, palliative care, geriatric medicine or other specialists may be beneficial (53). Complex palliative care interventions are necessary. Research showed that palliative care has positive effects on the care of people with dementia (136). However, evidence of the effectiveness of palliative care interventions for advanced dementia is scarce, there are still very few powered and randomized controlled trials on palliative interventions in advanced dementia and trials are often at risk for bias (9). To date trials have examined the effectiveness of interventions on enhancing decision making and advance care planning to improve care outcomes and reduce hospital and emergency transitions (137), the effectiveness of earlier involvement of palliative care to reduce transitions at the end of life (138) or special programs involving family carers and volunteers in the care (139).

Recommendations for person-centered care and continuity in carers and care setting (105) contribute maximizing comfort. From previous focus on deficits emphasis was put on an empathic approach understanding the individual’s experiences and help maintain personhood and relationship (140) Person-centered care for people with dementia can reduce neuropsychiatric symptoms, agitation and depression with stronger evidence for less advanced stages (141). Personhood is still essential in the advanced stages of dementia and may be achieved in various daily situations of care (74). Interventions based on person-centered care may be developed to improve quality of life. To integrate validation (142) and a method of sensory stimulus (143) are possible strategies to enhance person-centered care in the advanced stages of the disease. Another central issues for palliative care in dementia is to ensure continuity of care (105), referring to continuity of care setting and caregivers. Care transitions at the end-of-life are associated with burden and less quality of life. Hospital admission in nursing home residents with advanced dementia is common in the event of disease complications with questionable benefit due to distress of burdensome interventions and an unknown environment. Referrals to hospital were most common in the last three months with a slight decrease over years of burdensome care transitions in the last months and days of life (5, 144).

Comfort and quality of life are often care goals of palliative treatment for dementia. Quality of life is a goal of many interventions and it is often used to illustrate the effects of interventions, mostly as proxy evaluation (145). Quality of life is described as a multidimensional construct that includes various dimensions, such as physical and mental health, social support, life satisfaction, and providing comfort through symptom relieve (146). Instruments for assessing reliable quality of life and symptoms in advanced dementia depend on observations (147). Most instruments were developed for the phases of early and moderate dementia. For severe dementia the instruments Quality of life in dementia scale (QUALIDEM) (148) and Quality of life in late-stage dementia scale (QUALID) (100) can be used. QUALIDEM was developed as a self-reporting scale. The survey for people with advanced dementia is conducted as a proxy assessment with less validity than the self-disclosure scale. QUALID was developed for the target group severe dementia and translated into several languages. More complex and time-consuming instruments are also used to assess quality of life (140, 149).

## Discussion

The relevance for palliative care in dementia has been confirmed by clinical practice and research especially in the advanced stages. People with advanced dementia suffer from burdensome symptoms and complications. They have various needs and benefit from palliative care to ease their symptom burden and improve quality of life at the end of life.

Taking into consideration length of the time from diagnosis to death or duration of the advanced stages it underlines the recommendations for early integration of palliative care. At different points over the course of the disease whenever the goal is to decrease symptom burden and enhance quality of life. To foster acknowledging dementia as a life threatening syndrome is prerequisite. Health care professionals are required to give consideration to initiating palliative care. Coordinating care with one responsible professional carer during the course of the disease (150) may enhance continuity of care. These practices have yet to be established in the different health care systems. A care and support plan may be beneficial (150). A treatment plan referring to goals of care and formulated strategies for action in situation of emergency and acute complications may enhance interdisciplinary and interprofessional collaboration for effective treatment.

Nursing home are often the homes of many people with advanced dementia and as common places of care these institutions need to be supported for them to provide good palliative care. Health care professionals need to be qualified to deal with issues of care in advanced dementia. In home care people with advanced dementia seem to be under represented or may have little access to palliative care as several studies struggled recruiting people with dementia living at home (53, 74). Access to hospice care is still limited and needs to be increased. Health professionals in hospice care can provide care to relieve symptom burden and enhance quality of life, e.g., pain management. As many hospice and palliative home care teams are focused in cancer care they need to be skilled for specifics in dementia care, e.g., communication and symptom assessment. Establishing hospice and palliative care in nursing homes and collaboration with specialized palliative care services may meet needs of many people with advance dementia not having access to hospice. People with advanced dementia suffer burdensome symptoms and needs. Symptom management is important to increase quality of life and is still needs improvement. Raising awareness for common symptoms as pain, breathlessness or neuropsychiatric symptoms serves as basis for assessment and treatment.

Recognizing approaching end of life is important to provide good palliative care during this period. Tools may help to better estimate remaining life time and identify people at the end of life and can serve as starting point to reflect on appropriate goal of care. As nursing home staff recognized changes near death integrating systematically health professionals’ observations and experience offer an opportunity improving awareness for approaching death. Enhancing health professionals to reflect on their perception regularly may help to identify people with dementia in the last phase of life even earlier and change care goals if appropriate. Person-centered care and continuity of care is recommended for people with dementia. A person-centered approach may help to individualize care according to current symptoms and needs (105).

To maintain autonomy and see that the values and preferences of the person with dementia are implemented, advance care planning is beneficial. Various recommendations have been made to install advance care planning (107) though best time of initiating and approach remains unsolved. It is a very personal issue involving individual’s values, relationships, emotional, ethical, and legal aspects. Whenever implementing advance care planning the willingness of patients, their relatives and health care professionals must be considered individually (107). ACP is always an option for people with dementia and their family member, whereby they can make their decision to use this opportunity or not. Despite planning ahead and preparing advanced directives, unpredictable situations may occur and there may be no indicators as to how to deal with everyday matters (103). ACP can prevent burdensome interventions at the end of life, e.g., transitions from nursing home to hospital or invasive treatment. Still, it is important how health professionals react to acute events if they feel able to provide adequate care and feel comfortable with conservation at end of life (151).

In all settings, family members remain important as providers of care and care recipients. Caring for a family member with dementia can be a positive experience but is also associated with caregiver burden asfamily carers experience a range of burden through the whole course of dementia. At different points, e.g., diagnosis, transition to nursing home, proxy decision making they may need different means of support (120). To provide information on the course of dementia and possible complications is important to prepare for the end of life. In comparison to the early stage of the disease less is known about differences in caregiver burden and needs of family carers of people in the advanced stages of dementia. Prominent needs and burden may vary accordingly to different dementia stage, e.g., physical strain, financial burden, stress between caregiving and occupational duties. Some family carers are not recorded as carers, after death health care use and prescription of antidepressants, hypnotics, and anxiolytics increased (152). Family carers may benefit from palliative care in home care and nursing homes either as care providers or care recipients. To implement patients’ wish to die at home people with dementia and their caregivers are in need of palliative care and additional supporting services.

## Summary and Conclusion

- Dementia is a life-limiting syndrome with uncertainty in the course of the disease in the individual and challenges to predicting survival after diagnosis and approaching end of life. Health care providers should recognize signs of approaching death, then to provide comfort care for the person with advanced dementia and the family carers.
- People with advanced dementia have complex health care needs and benefit from palliative care interventions bringing symptom relief and comfort. Integration of palliative care early in the course of the disease may prevent arduous interventions.
- Burdensome symptoms often accompany advanced dementia, thus regular assessment of symptoms and domains of quality of life using appropriate instruments and careful observation are essential for ensuring comfort.
- The appropriateness of non-pharmacological interventions should be considered given the high number of associated psychosocial needs and neuropsychiatric symptoms. Interventions based on person-centered care may also prove beneficial.
- Recurring treatment decisions are necessary throughout the course of the disease preferably involving all significant stakeholders. The offer of advance care planning is essential for providing comfort and avoiding burdensome interventions at the end of life.
- Family carers experience distress and require support across the entire course of the disease. They need information about what to expect at the end of life with dementia and support with proxy decision making.
- Nursing homes and hospitals are common places of death where people with dementia have low access to hospice care. Long-term care facilities should be enabled to provide good palliative care until the end of life, avoiding burdensome transitions at that point while offering continuity of care.
- There remains a need for evidenced based interventions to evaluatethe effectiveness of palliative care in advanced dementia. Interprofessional and interdisciplinary collaborations among (old age) psychiatrists, geriatricians, general practitioners, palliative care teams (or others) should be considered to best manage the complex care needs of people with dementia.

## Author Contributions

YE and KP drafted and finalized the manuscript. HG, HS, and RV designed and revised manuscript.

## Conflict of Interest

The authors declare that the research was conducted in the absence of any commercial or financial relationships that could be construed as a potential conflict of interest.

## References

[1] Prince M Ali GC Guerchet M Prina AM Albanese E Wu YT Recent global trends in the prevalence and incidence of dementia, and survival with dementia. Alzheimer’s Res Ther (2016) 8(1):23. 10.1186/s13195-016-0188-8 27473681 PMC4967299

[2] World Health Organization Dementia: a public health priority. United Kingdom: World Health Organization (2012).

[3] World Health Organization WHO Definition of Palliative Care 2002. (2002) http://www.who.int/cancer/palliative/definition/en/.

[4] Etkind SN Bone AE Gomes B Lovell N Evans CJ Higginson IJ How many people will need palliative care in 2040? Past trends, future projections and implications for services. BMC Med (2017) 15(1):102. 10.1186/s12916-017-0860-2 28514961 PMC5436458

[5] Mitchell SL Teno JM Kiely DK Shaffer ML Jones RN Prigerson HG The clinical course of advanced dementia. New Engl J Med (2009) 361(16):1529–38. 10.1056/NEJMoa0902234 PMC2778850 19828530

[6] Moens K Higginson IJ Harding R Are there differences in the prevalence of palliative care-related problems in people living with advanced cancer and eight non-cancer conditions? A systematic review. J Pain Symptom Manage (2014) 48(4):660–77. 10.1016/j.jpainsymman.2013.11.009 24801658

[7] van Soest-Poortvliet MC van der Steen JT de Vet HC Hertogh CM Deliens L Onwuteaka-Philipsen BD Comfort goal of care and end-of-life outcomes in dementia: A prospective study. Palliat Med (2015) 29(6):538–46. 10.1177/0269216315570409 25690602

[8] Perrar KM Voltz R Palliativversorgung. In: Jessen F, editor. Handbuch Alzheimer-Krankheit Grundlagen - Diagnostik - Therapie - Versorgung - Prävention. Berlin: De Gruyter (2018). p. 528–37.

[9] Murphy E Froggatt K Connolly S O’Shea E Sampson EL Casey D Palliative care interventions in advanced dementia. Cochrane Database Syst Rev (2016) 12:Cd011513. 10.1002/14651858.CD011513.pub2 27911489 PMC6463843

[10] Pleschberger S Palliative Care und Dementia Care – Gemeinsamkeiten und Unterschiede zweier innovativer Versorgungskonzepte im Lichte der Entwicklung in Deutschland. Pflege&Gesellschaft (2014) 19(3):197–267.

[11] Lunney JR Lynn J Foley DJ Lipson S Guralnik JM Patterns of functional decline at the end of life. JAMA (2003) 289(18):2387–92. 10.1001/jama.289.18.2387 12746362

[12] Morris JC The Clinical Dementia Rating (CDR): current version and scoring rules. Neurology (1993) 43(11):2412–4. 10.1212/WNL.43.11.2412-a 8232972

[13] Reisberg B Ferris SH de Leon MJ Crook T Global Deterioration Scale (GDS). Psychopharmacol Bull (1988) 24(4):661–3. 3249768

[14] Sampson EL Palliative care for people with dementia. Br Med Bull (2010) 96(1):159–74. 10.1093/bmb/ldq024 20675657

[15] Kuehlmeyer K Schuler AF Kolb C Borasio GD Jox RJ Evaluating Nonverbal Behavior of Individuals with Dementia During Feeding: A Survey of the Nursing Staff in Residential Care Homes for Elderly Adults. J Am Geriatr Soc (2015) 63(12):2544–9. 10.1111/jgs.13822 26566872

[16] Eisenmann Y Schmidt H Raymond V Perrar KM Expression and Perception of Needs in Severe Dementia: A Case Study. J Hosp Palliat Nurs (2016) 18(3):268–72. 10.1097/NJH.0000000000000245

[17] Diehl-Schmid J Riedl L Rusing U Hartmann J Bertok M Levin C [Provision of palliative care for people with advanced dementia]. Nervenarzt (2018) 89(5):524–9. 10.1007/s00115-017-0468-y 29327100

[18] Nowossadeck S Engstler H Klaus D Pflege und Unterstützung durch Angehörige. Berlin: Deutsches Zentrum für Altersfragen (2016). Contract No.: Heft 1/2016.

[19] Prince M Knapp M Guerchet M McCrone P Prina M Comas-Herrera A Dementia UK: Update. Alzheimer's Society (2014).

[20] Schäufele M Köhler L Hendlmeier I Hoell A Weyerer S Prävalenz von Demenzen und ärztliche Versorgung in deutschen Pflegeheimen: eine bundesweite repräsentative Studie. Psychiat Prax (2013) 40(4):200–6. 10.1055/s-0033-1343141 23670714

[21] Arrighi HM Neumann PJ Lieberburg IM Townsend RJ Lethality of Alzheimer disease and its impact on nursing home placement. Alzheimer Dis Assoc Disord (2010) 24(1):90–5. 10.1097/WAD.0b013e31819fe7d1 19568155

[22] Mitchell SL Black BS Ersek M Hanson LC Miller SC Sachs GA Advanced dementia: state of the art and priorities for the next decade. Ann Internal Med (2012) 156:45–51. 10.7326/0003-4819-156-1-201201030-00008 22213494 PMC3261500

[23] Reyniers T Deliens L Pasman HR Morin L Addington-Hall J Frova L International variation in place of death of older people who died from dementia in 14 European and non-European countries. J Am Med Dir Assoc (2015) 16(2):165–71. 10.1016/j.jamda.2014.11.003 25544001

[24] Afram B Stephan A Verbeek H Bleijlevens MH Suhonen R Sutcliffe C Reasons for institutionalization of people with dementia: informal caregiver reports from 8 European countries. J Am Med Dir Assoc (2014) 15(2):108–16. 10.1016/j.jamda.2013.09.012 24238605

[25] Stock S Ihle P Simic S Rupprecht C Schubert I Lappe V Prävalenz von Demenz bei Versicherten mit und ohne deutsche Staatsangehörigkeit. Bundesgesundheitsblatt - Gesundheitsforschung - Gesundheitsschutz (2018) 61(4):404–11. 10.1007/s00103-018-2711-5 29487974

[26] Escobar Pinzon L Claus M Perrar K Zepf K Dying with dementia: symptom burden, quality of care, and place of death. Dtsch Arztebl Int (2013) 110(12):195–202. 10.3238/arztebl.2013.0195 23589742 PMC3622236

[27] Badrakalimuthu V Barclay S Do people with dementia die at their preferred location of death? A systematic literature review and narrative synthesis. Age Ageing (2014) 43(1):13–9. 10.1093/ageing/aft151 24128594

[28] Dasch B Bausewein C Feddersen B Place of death in patients with dementia and the association with comorbidities: a retrospective population-based observational study in Germany. BMC Palliat Care (2018) 17(1):80. 10.1186/s12904-018-0334-0 29793476 PMC5966860

[29] Nakanishi M Nakashima T Shindo Y Niimura J Nishida A Japanese Care Location and Medical Procedures for People with Dementia in the Last Month of Life. J Alzheimers Dis (2016) 51(3):747–55. 10.3233/JAD-150898 26890762

[30] Fischer S Bosshard G Zellweger U Faisst K Der Sterbeort: “Wo sterben die Menschen heute in der Schweiz?”. Z Gerontol Geriatr (2004) 37(6):467–74. 10.1007/s00391-004-0216-3 15614599

[31] Houttekier D Cohen J Bilsen J Addington-Hall J Onwuteaka-Philipsen BD Deliens L Place of death of older persons with dementia. A study in five European countries. J Am Geriatr Soc (2010) 58(4):751–6. 10.1111/j.1532-5415.2010.02771.x 20398157

[32] Mitchell SL Teno JM Miller SC Mor V A national study of the location of death for older persons with dementia. J Am Geriatr Soc (2005) 53(2):299–305. 10.1111/j.1532-5415.2005.53118.x 15673356

[33] Goluke NMS van de Vorst IE Vaartjes IH Geerlings MI de Jonghe A Bots ML Risk factors for in-hospital mortality in patients with dementia. Maturitas (2019) 129:57–61. 10.1016/j.maturitas.2019.08.007 31547914

[34] Wiggins N Droney J Mohammed K Riley J Sleeman KE Understanding the factors associated with patients with dementia achieving their preferred place of death: a retrospective cohort study. Age Ageing (2019) 48(3):433–9. 10.1093/ageing/afz015 PMC6503933 30806452

[35] Cross SH Kaufman BG Warraich H Trends And Factors Associated With Place Of Death Among Individuals With Cardiovascular Disease In The United States. Innovation Aging (2019) 3:132–S3. 10.1093/geroni/igz038.482

[36] Ankuda CK Mitchell SL Gozalo P Mor V Meltzer D Teno JM Association of Physician Specialty with Hospice Referral for Hospitalized Nursing Home Patients with Advanced Dementia. J Am Geriatr Soc (2017) 65(8):1784–8. 10.1111/jgs.14888 PMC5555793 28369754

[37] Bickel H Die Häufigkeit von Demenzerkrankungen Berlin: Deutsche Alzheimer Gesellschaft. Deutsche Alzheimer Gesellschaft (2016). Available at: https://www.deutsche-alzheimer.de/fileadmin/alz/pdf/factsheets/infoblatt1_haeufigkeit_demenzerkrankungen_dalzg.pdf.

[38] Niu H Alvarez-Alvarez I Guillen-Grima F Al-Rahamneh MJ Aguinaga-Ontoso I Trends of mortality from Alzheimer’s disease in the European Union, 1994-2013. Eur J Neurol (2017) 24(6):858–66. 10.1111/ene.13302 28544405

[39] O’Dowd A Dementia is now leading cause of death in women in England. Bmj (2017) 358:j3445. 10.1136/bmj.j3445 28710087

[40] Murphy SL Xu J Kochanek KD Arias E Mortality in the United States, 2017. NCHS Data Brief (2018) (328):1–8. 30500322

[41] Koller D Kaduszkiewicz H van den Bussche H Eisele M Wiese B Glaeske G Survival in patients with incident dementia compared with a control group: a five-year follow-up. Int Psychogeriatr (2012) 24(9):1522–30. 10.1017/S1041610212000361 22458976

[42] Haaksma ML Eriksdotter M Rizzuto D Leoutsakos J-MS Olde Rikkert MGM Melis RJF Survival time tool to guide care planning in people with dementia. Neurology (2020) 94(5):e538–e48. 10.1212/WNL.0000000000008745 PMC7080282 31843808

[43] Zanetti O Solerte SB Cantoni F Life expectancy in Alzheimer’s disease (AD). Arch Gerontol Geriatr (2009) 49 Suppl 1:237–43. 10.1016/j.archger.2009.09.035 19836639

[44] Brodaty H Seeher K Gibson L Dementia time to death: a systematic literature review on survival time and years of life lost in people with dementia. Int Psychogeriatr (2012) 24(7):1034–45. 10.1017/S1041610211002924 22325331

[45] Garcia-Ptacek S Kareholt I Cermakova P Rizzuto D Religa D Eriksdotter M Causes of Death According to Death Certificates in Individuals with Dementia: A Cohort from the Swedish Dementia Registry. J Am Geriatr Soc (2016) 64(11):e137–e42. 10.1111/jgs.14421 27801938

[46] Strand BH Knapskog AB Persson K Edwin TH Amland R Mjorud M Survival and years of life lost in various aetiologies of dementia, mild cognitive impairment (MCI) and subjective cognitive decline (SCD) in Norway. PloS One (2018) 13(9):e0204436. 10.1371/journal.pone.0204436 30240425 PMC6150521

[47] Rizzuto D Bellocco R Kivipelto M Clerici F Wimo A Fratiglioni L Dementia after age 75: survival in different severity stages and years of life lost. Curr Alzheimer Res (2012) 9(7):795–800. 10.2174/156720512802455421 22299618

[48] Aworinde J Werbeloff N LG Livingston G Sommerlad A Dementia severity at death: a register-based cohort study. BMC Psychiatry (2018) 18(1):355. 10.1186/s12888-018-1930-5 30382865 PMC6211473

[49] Koopmans RT Ekkerink JL van Weel C Survival to late dementia in Dutch nursing home patients. J Am Geriatr Soc (2003) 51(2):184–7. 10.1046/j.1532-5415.2003.51056.x 12558714

[50] Diehl-Schmid J Pohl C Perneczky R Hartmann J Forstl H Kurz A [Initial symptoms, survival and causes of death in 115 patients with frontotemporal lobar degeneration]. Fortschr Neurol-Psychiatr (2007) 75(12):708–13. 10.1055/s-2006-932201 16972211

[51] Koopmans RT van der Sterren KJ van der Steen JT The ‘natural’ endpoint of dementia: death from cachexia or dehydration following palliative care? Int J Geriatr Psychiatry (2007) 22(4):350–5. 10.1002/gps.1680 17022107

[52] Klapwijk MS Caljouw MA van Soest-Poortvliet MC van der Steen JT Achterberg WP Symptoms and treatment when death is expected in dementia patients in long-term care facilities. BMC Geriatr (2014) 14:99. 10.1186/1471-2318-14-99 25181947 PMC4158395

[53] Sampson EL Candy B Davis S Gola AB Harrington J King M Living and dying with advanced dementia: A prospective cohort study of symptoms, service use and care at the end of life. Palliat Med (2018) 32(3):668–81. 10.1177/0269216317726443 PMC5987852 28922625

[54] Koppitz A Bosshard G Schuster DH Hediger H Imhof L Type and course of symptoms demonstrated in the terminal and dying phases by people with dementia in nursing homes. Z Gerontol Geriatr (2015) 48(2):176–83. 10.1007/s00391-014-0668-z 25119700

[55] Hendriks SA Smalbrugge M Hertogh CM van der Steen JT Dying with dementia: symptoms, treatment, and quality of life in the last week of life. J Pain Symptom Manage (2014) 47(4):710–20. 10.1016/j.jpainsymman.2013.05.015 23916680

[56] Vandervoort A van den Block L van der Steen JT Volicer L Vander Stichele R Houttekier D Nursing home residents dying with dementia in Flanders, Belgium: A nationwide postmortem study on clinical characteristics and quality of dying. J Am Med Dir Assoc (2013) 14(7):485–92. 10.1016/j.jamda.2013.01.016 23523319

[57] Jang H Kim JH Choi SH Lee Y Hong CH Jeong JH Body Mass Index and Mortality Rate in Korean Patients with Alzheimer’s Disease. J Alzheimers Dis (2015) 46(2):399–406. 10.3233/JAD-142790 25737040

[58] Brown MA Sampson EL Jones L Barron AM Prognostic indicators of 6-month mortality in elderly people with advanced dementia: a systematic review. Palliat Med (2013) 27(5):389–400. 10.1177/0269216312465649 23175514 PMC3652641

[59] Mitchell SL Miller SC Teno JM Davis RB Shaffer ML The Advanced Dementia Prognostic Tool (ADEPT): A Risk Score to Estimate Survival in Nursing Home Residents with Advanced Dementia. J Pain Symptom Manage (2010) 40(5):639–51. 10.1016/j.jpainsymman.2010.02.014 PMC2981683 20621437

[60] Mitchell SL Kiely DK Hamel M Park PS Morris JN Fries BE Estimating prognosis for nursing home residents with advanced dementia. JAMA (2004) 291(22):2734–40. 10.1001/jama.291.22.2734 15187055

[61] Mitchell SL Kiely DK Hamel MB Dying with advanced dementia in the nursing home. Arch Internal Med (2004) 164(3):321–6. 10.1001/archinte.164.3.321 14769629

[62] van der Steen JT Deliens L Koopmans RTCM Onwuteaka-Philipsen BD Physicians’ perceptions of suffering in people with dementia at the end of life. Palliat Support Care (2017) 15(5):587–99. 10.1017/S1478951516000985 28112072

[63] Loizeau AJ Shaffer ML Habtemariam DA Hanson LC Volandes AE Mitchell SL Association of Prognostic Estimates With Burdensome Interventions in Nursing Home Residents With Advanced Dementia. JAMA Internal Med (2018) 178(7):922–9. 10.1001/jamainternmed.2018.1413 PMC6033677 29813159

[64] Aminoff BZ Mini Suffering State Examination (MSSE) Scale: first 5 years. Israel Med Assoc J IMAJ (2004) 6(10):645–6. 15473601

[65] Thomas K The Gold Standards Framework Proactive Identification Guidance (PIG). 6 ed The Gold Standards Framework Centre in End of Life Care (2016).

[66] Gómez-Batiste X Martínez-Muñoz M Blay C Amblàs J Vila L Costa X Utility of the NECPAL CCOMS-ICO(©) tool and the Surprise Question as screening tools for early palliative care and to predict mortality in patients with advanced chronic conditions: A cohort study. Palliat Med (2017) 31(8):754–63. 10.1177/0269216316676647 27815556

[67] Soares LGL Japiassu AM Gomes LC Pereira R Pecanha C Goldgaber T Prevalence and intensity of dyspnea, pain, and agitation among people dying with late stage dementia compared with people dying with advanced cancer: a single-center preliminary study in Brazil. Ann Palliat Med (2018) 7(4):437–43. 10.21037/apm.2018.05.06 29860860

[68] Erdal A Flo E Selbaek G Aarsland D Bergh S Slettebo DD Associations between pain and depression in nursing home patients at different stages of dementia. J Affect Disord (2017) 218:8–14. 10.1016/j.jad.2017.04.038 28456075

[69] Malara A De Biase GA Bettarini F Ceravolo F Di Cello S Garo M Pain Assessment in Elderly with Behavioral and Psychological Symptoms of Dementia. J Alzheimers Dis (2016) 50(4):1217–25. 10.3233/JAD-150808 PMC4927851 26757042

[70] Husebo BS Ballard C Aarsland D Pain treatment of agitation in patients with dementia: a systematic review. Int J Geriatr Psychiatry (2011) 26(10):1012–8. 10.1002/gps.2649 21308784

[71] Burns M McIlfatrick S Palliative care in dementia: literature review of nurses’ knowledge and attitudes towards pain assessment. Int J Palliat Nurs (2015) 21(8):400–7. 10.12968/ijpn.2015.21.8.400 26312536

[72] Warden V Hurley AC Volicer L Development and Psychometric Evaluation of the Pain Assessment in Advanced Dementia (PAINAD) Scale. J Am Med Dir Assoc (2003) 4:9–15. 10.1097/01.JAM.0000043422.31640.F7 12807591

[73] van Kooten J Smalbrugge M van der Wouden JC Stek ML Hertogh C Prevalence of Pain in Nursing Home Residents: The Role of Dementia Stage and Dementia Subtypes. J Am Med Dir Assoc (2017) 18(6):522–7. 10.1016/j.jamda.2016.12.078 28236607

[74] Schmidt H Eisenmann Y Golla H Voltz R Perrar KM Needs of people with advanced dementia in their final phase of life: A multi-perspective qualitative study in nursing homes. Palliat Med (2018) 32(3):657–67. 10.1177/0269216317746571 29235393

[75] Hendriks SA Smalbrugge M Galindo-Garre F Hertogh CM van der Steen JT From admission to death: prevalence and course of pain, agitation, and shortness of breath, and treatment of these symptoms in nursing home residents with dementia. J Am Med Dir Assoc (2015) 16(6):475–81. 10.1016/j.jamda.2014.12.016 25736822

[76] Schneider J Algharably E Budnick A Wenzel A Drager D Kreutz R Deficits in pain medication in older adults with chronic pain receiving home care: A cross-sectional study in Germany. PloS One (2020) 15(2):e0229229. 10.1371/journal.pone.0229229 32084203 PMC7034806

[77] Husebo BS Ballard C Fritze F Sandvik RK Aarsland D Efficacy of pain treatment on mood syndrome in patients with dementia: a randomized clinical trial. Int J Geriatr Psychiatry (2014) 29(8):828–36. 10.1002/gps.4063 24806873

[78] Achterberg WP Pieper MJ van Dalen-Kok AH de Waal MW Husebo BS Lautenbacher S Pain management in patients with dementia. Clin Interventions Aging (2013) 8:1471–82. 10.2147/CIA.S36739 PMC3817007 24204133

[79] van der Maarel-Wierink CD Vanobbergen JN Bronkhorst EM Schols JM de Baat C Risk factors for aspiration pneumonia in frail older people: a systematic literature review. J Am Med Dir Assoc (2011) 12(5):344–54. 10.1016/j.jamda.2010.12.099 21450240

[80] Mitchell SL Care of patients with advanced dementia. N Engl J Med (2015) 372(5):2533–40. 10.1056/NEJMcp1412652 PMC4539157 26107053

[81] Bedi R Dementia and oral health. J Public Health Policy (2015) 36(1):128–30. 10.1057/jphp.2014.49 25563169

[82] Delwel S Binnekade TT Perez R Hertogh C Scherder EJA Lobbezoo F Oral hygiene and oral health in older people with dementia: a comprehensive review with focus on oral soft tissues. Clin Oral Investig (2018) 22(1):93–108. 10.1007/s00784-017-2264-2 PMC5748411 29143189

[83] Delwel S Binnekade TT Perez RS Hertogh CM Scherder EJ Lobbezoo F Oral health and orofacial pain in older people with dementia: a systematic review with focus on dental hard tissues. Clin Oral Investig (2017) 21(1):17–32. 10.1007/s00784-016-1934-9 PMC5203832 27631597

[84] Bartholomeyczik S [Prevention of malnutrition in institutional long-term care with the DNQP “nutrition management” expert standard]. Bundesgesundheitsblatt Gesundheitsforschung Gesundheitsschutz (2019) 62(3):304–10. 10.1007/s00103-019-02878-1 30671604

[85] Teno JM Gozalo PL Mitchell SL Kuo S Rhodes RL Bynum JP Does feeding tube insertion and its timing improve survival? J Am Geriatr Soc (2012) 60(10):1918–21. 10.1111/j.1532-5415.2012.04148.x PMC3470758 23002947

[86] Ayman AR Khoury T Cohen J Chen S Yaari S Daher S PEG Insertion in Patients With Dementia Does Not Improve Nutritional Status and Has Worse Outcomes as Compared With PEG Insertion for Other Indications. J Clin Gastroenterol (2017) 51(5):417–20. 10.1097/MCG.0000000000000624 27505401

[87] Givens JL Selby K Goldfeld KS Mitchell SL Hospital transfers of nursing home residents with advanced dementia. J Am Geriatr Soc (2012) 60(5):905–9. 10.1111/j.1532-5415.2012.03919.x PMC3354640 22428661

[88] Manabe T Fujikura Y Mizukami K Akatsu H Kudo K Pneumonia-associated death in patients with dementia: A systematic review and meta-analysis. PloS One (2019) 14(3):e0213825. 10.1371/journal.pone.0213825 30870526 PMC6417730

[89] Givens JL Jones RN Shaffer ML Kiely DK Mitchell SL Survival and comfort after treatment of pneumonia in advanced dementia. Arch Intern Med (2010) 170(13):1102–7. 10.1001/archinternmed.2010.181 PMC2914628 20625013

[90] van der Maaden T de Vet HC Achterberg WP Boersma F Schols JM Mehr DR Improving comfort in people with dementia and pneumonia: a cluster randomized trial. BMC Med (2016) 14(1):116. 10.1186/s12916-016-0663-x 27515720 PMC4981997

[91] Campbell ML Kiernan JM Strandmark J Yarandi HN Trajectory of Dyspnea and Respiratory Distress among Patients in the Last Month of Life. J Palliat Med (2018) 21(2):194–9. 10.1089/jpm.2017.0265 28817366

[92] Campbell ML Templin T Walch J A Respiratory Distress Observation Scale for patients unable to self-report dyspnea. J Palliat Med (2010) 13(3):285–90. 10.1089/jpm.2009.0229 20078243

[93] Booth S Burkin J Moffat C Spathis A Managing Breathlessness in Clinical Practice. London: Springer (2014). p. 284.

[94] Lyketsos CG Carrillo MC Ryan JM Khachaturian AS Trzepacz P Amatniek J Neuropsychiatric symptoms in Alzheimer’s disease. Alzheimer’s Dement J Alzheimer’s Assoc (2011) 7(5):532–9. 10.1016/j.jalz.2011.05.2410 PMC3299979 21889116

[95] Cohen-Mansfield J Dakheel-Ali M Marx MS Thein K Regier NG Which unmet needs contribute to behavior problems in persons with advanced dementia? Psychiatry Res (2015) 228(1):59–64. 10.1016/j.psychres.2015.03.043 25933478 PMC4451402

[96] Cummings JL Mega M Gray K Rosenberg-Thompson S Carusi DA Gornbein J The Neuropsychiatric Inventory: comprehensive assessment of psychopathology in dementia. Neurology (1994) 44(12):2308–14. 10.1212/WNL.44.12.2308 7991117

[97] Hewer W Holthoff-Detto V Palliativmedizinische Aspekte der Versorgung von Menschen mit Demenz und Delir. Der Nervenarzt (2020) 91(5):398–403. 10.1007/s00115-020-00884-2 32123935

[98] Agar MR Lawlor PG Quinn S Draper B Caplan GA Rowett D Efficacy of Oral Risperidone, Haloperidol, or Placebo for Symptoms of Delirium Among Patients in Palliative Care: A Randomized Clinical Trial. JAMA Internal Med (2017) 177(1):34–42. 10.1001/jamainternmed.2016.7491 27918778

[99] Anderson AR Deng J Anthony RS Atalla SA Monroe TB Using Complementary and Alternative Medicine to Treat Pain and Agitation in Dementia: A Review of Randomized Controlled Trials from Long-Term Care with Potential Use in Critical Care. Crit Care Nurs Clin North Am 29(4):519–37. 10.1016/j.cnc.2017.08.010 PMC5687304 29107312

[100] Weiner MF Martin-Cook K Svetlik DA Saine K Foster B Fontaine CS The quality of life in late-stage dementia (QUALID) scale. J Am Med Dir Assoc (2000) 1(3):114–6. 10.1037/t00432-000 12818023

[101] Meagher D Agar MR Teodorczuk A Debate article: Antipsychotic medications are clinically useful for the treatment of delirium. Int J Geriatr Psychiatry (2018) 33(11):1420–7. 10.1002/gps.4759 28758323

[102] Bush SH Lawlor PG Ryan K Centeno C Lucchesi M Kanji S Delirium in adult cancer patients: ESMO Clinical Practice Guidelines. Ann Oncol Off J Eur Soc Med Oncol (2018) 29(Suppl 4):iv143–iv65. 10.1093/annonc/mdy147 29992308

[103] Bosisio F Jox RJ Jones L Rubli Truchard E Planning ahead with dementia: what role can advance care planning play? A review on opportunities and challenges. Swiss Med Weekly (2018) 148:w14706. 10.4414/smw.2018.14706 30594990

[104] Jox RJ Lost decisional capacity – lost chance of Advance Care Planning? Bioeth Forum (2016) 9(3):109–10.

[105] van der Steen JT Radbruch L Hertogh CM de Boer ME Hughes JC Larkin P White paper defining optimal palliative care in older people with dementia: a Delphi study and recommendations from the European Association for Palliative Care. Palliat Med (2014) 28(3):197–209. 10.1177/0269216313493685 23828874

[106] Ryan T Amen KM McKeown J The advance care planning experiences of people with dementia, family caregivers and professionals: a synthesis of the qualitative literature. Ann Palliat Med (2017) 6(4):380–9. 10.21037/apm.2017.06.15 28754049

[107] Harrison Dening K Sampson EL De Vries K Advance care planning in dementia: recommendations for healthcare professionals. Palliat Care (2019) 12:1–10. 10.1177/1178224219826579 PMC6393818 30833812

[108] Brazil K Carter G Cardwell C Clarke M Hudson P Froggatt K Effectiveness of advance care planning with family carers in dementia nursing homes: A paired cluster randomized controlled trial. Palliat Med (2018) 32(3):603–12. 10.1177/0269216317722413 28786323

[109] Ernecoff NC Wessell KL Hanson LC Dusetzina SB Shea CM Weinberger M Elements of Palliative Care in the Last 6 Months of Life: Frequency, Predictors, and Timing. J Gen Internal Med (2019) 35(3):753–61. 10.1007/s11606-019-05349-0 PMC7080876 31650402

[110] Gozalo P Teno JM Mitchell SL Skinner J Bynum J Tyler D End-of-life transitions among nursing home residents with cognitive issues. N Engl J Med (2011) 365(13):1212–21. 10.1056/NEJMsa1100347 PMC3236369 21991894

[111] Vandervoort A Houttekier D Van den Block L van der Steen JT Vander Stichele R Deliens L Advance care planning and physician orders in nursing home residents with dementia: a nationwide retrospective study among professional caregivers and relatives. J Pain Symptom Manage (2014) 47(2):245–56. 10.1016/j.jpainsymman.2013.03.009 23796587

[112] Robinson L Dickinson C Rousseau N Beyer F Clark A Hughes J A systematic review of the effectiveness of advance care planning interventions for people with cognitive impairment and dementia. Age Ageing (2012) 41(2):263–9. 10.1093/ageing/afr148 PMC3280677 22156555

[113] Lee RY Brumback LC Sathitratanacheewin S Lober WB Modes ME Lynch YT Association of Physician Orders for Life-Sustaining Treatment With ICU Admission Among Patients Hospitalized Near the End of Life. Jama (2020) 323(10):950–60. 10.1001/jama.2019.22523 PMC7042829 32062674

[114] Sellars M Chung O Nolte L Tong A Pond D Fetherstonhaugh D Perspectives of people with dementia and carers on advance care planning and end-of-life care: A systematic review and thematic synthesis of qualitative studies. Palliat Med (2019) 33(3):274–90. 10.1177/0269216318809571 PMC6376607 30404576

[115] Volhard T Jessen F Kleineidam L Wolfsgruber S Lanzerath D Wagner M Advance directives for future dementia can be modified by a brief video presentation on dementia care: An experimental study. PloS One (2018) 13(5):e0197229. 10.1371/journal.pone.0197229 29795605 PMC5967707

[116] Scholzel-Dorenbos CJ Draskovic I Vernooij-Dassen MJ Olde Rikkert MG Quality of life and burden of spouses of Alzheimer disease patients. Alzheimer Dis Assoc Disord (2009) 23(2):171–7. 10.1097/WAD.0b013e318190a260 19484919

[117] Jansen S Weiß S Angehörigenarbeit. In: Jessen F, editor. Handbuch Alzheimer-Krankheit Grundlagen - Diagnostik - Therapie - Versorgung - Prävention. Berlin: De Gruyter (2018). p. 554–63.

[118] MarieCurieCancerCare Society As. Living and dying with dementia in England: Barriers to care. Marie Curie Cancer Care, and Alzheimer's Society (2014).

[119] Sachs GA Shega JW Cox-Hayley D Barriers to excellent end-of-life care for patients with dementia. J Gen Internal Med (2004) 19(10):1057–63. 10.1111/j.1525-1497.2004.30329.x PMC1492583 15482560

[120] D’Onofrio G Sancarlo D Addante F Ciccone F Cascavilla L Paris F Caregiver burden characterization in patients with Alzheimer’s disease or vascular dementia. Int J Geriatr Psychiatry (2015) 30(9):891–9. 10.1002/gps.4232 25475248

[121] Gaugler JE Yu F Krichbaum K Wyman JF Predictors of nursing home admission for persons with dementia. Med Care (2009) 47(2):191–8. 10.1097/MLR.0b013e31818457ce 19169120

[122] Moore KJ Davis S Gola A Harrington J Kupeli N Vickerstaff V Experiences of end of life amongst family carers of people with advanced dementia: longitudinal cohort study with mixed methods. BMC Geriatr (2017) 17(1):135. 10.1186/s12877-017-0523-3 28673257 PMC5496359

[123] Müller C Lautenschläger S Meyer G Stephan A Interventions to support people with dementia and their caregivers during the transition from home care to nursing home care: A systematic review. Int J Nurs Stud (2017) 71:139–52. 10.1016/j.ijnurstu.2017.03.013 28411508

[124] Armstrong MJ Alliance S Corsentino P DeKosky ST Taylor A Cause of Death and End-of-Life Experiences in Individuals with Dementia with Lewy Bodies. J Am Geriatr Soc (2019) 67(1):67–73. 10.1111/jgs.15608 30291740

[125] Zarit SH Reever KE Bach-Peterson J Relatives of the impaired elderly: correlates of feelings of burden. Gerontol (1980) 20(6):649–55. 10.1093/geront/20.6.649 7203086

[126] O’Malley KA Qualls SH Preliminary Evidence for the Validity and Reliability of the Caregiver Reaction Scale. Clin Gerontolt (2017) 40(4):281–94. 10.1080/07317115.2016.1198858 28452639

[127] Erel M Marcus EL Dekeyser-Ganz F Barriers to palliative care for advanced dementia: a scoping review. Ann Palliat Med (2017) 6(4):365–79. 10.21037/apm.2017.06.13 28754048

[128] Kupeli N Leavey G Moore K Harrington J Lord K King M Context, mechanisms and outcomes in end of life care for people with advanced dementia. BMC Palliat Care (2016) 15:31. 10.1186/s12904-016-0103-x 26965309 PMC4785626

[129] Davies N Maio L Vedavanam K Manthorpe J Vernooij-Dassen M Iliffe S Barriers to the provision of high-quality palliative care for people with dementia in England: a qualitative study of professionals’ experiences. Health Soc Care Commun (2014) 22(4):386–94. 10.1111/hsc.12094 PMC4265301 24372976

[130] Hill E Savundranayagam MY Zecevic A Kloseck M Staff Perspectives of Barriers to Access and Delivery of Palliative Care for Persons With Dementia in Long-Term Care. Am J Alzheimer’s Dis Other Dement (2018) 33(5):284–91. 10.1177/1533317518765124 PMC10852501 29554814

[131] Nakanishi M Nakashima T Shindo Y Miyamoto Y Gove D Radbruch L An evaluation of palliative care contents in national dementia strategies in reference to the European Association for Palliative Care white paper. Int Psychogeriatr (2015) 27(9):1551–61. 10.1017/S1041610215000150 25678323

[132] van der Steen JT Radbruch L de Boer ME Junger S Hughes JC Larkin P Achieving consensus and controversy around applicability of palliative care to dementia. Int Psychogeriatr (2016) 28(1):133–45. 10.1017/S1041610215000824 26060924

[133] Perrar KM Schmidt H Eisenmann Y Cremer B Voltz R Needs of people with severe dementia at the end-of-life: a systematic review. J Alzheimer’s Dis JAD (2015) 43(2):397–413. 10.3233/JAD-140435 25096623

[134] van der Roest HG Meiland FJ Maroccini R Comijs HC Jonker C Droes RM Subjective needs of people with dementia: a review of the literature. Int Psychogeriatr / IPA (2007) 19(3):559–92. 10.1017/S1041610206004716 17201993

[135] Hancock GA Woods B Challis D Orrell M The needs of older people with dementia in residential care. Int J Geriatr Psychiatry (2006) 21(1):43–9. 10.1002/gps.1421 16323258

[136] Singer AE Goebel JR Kim YS Dy SM Ahluwalia SC Clifford M Populations and Interventions for Palliative and End-of-Life Care: A Systematic Review. J Palliat Med (2016) 19(9):995–1008. 10.1089/jpm.2015.0367 27533892 PMC5011630

[137] Hanson LC Kistler CE Lavin K Gabriel SL Ernecoff NC Lin FC Triggered Palliative Care for Late-Stage Dementia: A Pilot Randomized Trial. J Pain Symptom Manage (2019) 57(1):10–9. 10.1016/j.jpainsymman.2018.10.494 PMC6310628 30342242

[138] Miller SC Lima JC Intrator O Martin E Bull J Hanson LC Specialty Palliative Care Consultations for Nursing Home Residents With Dementia. J Pain Symptom Manage (2017) 54(1):9–16.e5. 10.1016/j.jpainsymman.2017.03.005 28438589 PMC5663286

[139] Manzar B Volicer L Effects of Namaste Care: Pilot Study. Am J Alzheimer’s Dis (2015) 2(1):24–37. 10.7726/ajad.2015.1003

[140] Kitwood T Dementia Reconsidered. The person comes first. Buckingham; Bristol: Open University Press (1997).

[141] Kim SK Park M Effectiveness of person-centered care on people with dementia: a systematic review and meta-analysis. Clin Interventions Aging (2017) 12:381–97. 10.2147/CIA.S117637 PMC5322939 28255234

[142] Feil N Klerk-Rubin D Validation: Ein Weg zum Verständnis verwirrter alter Menschen Reinhardt Ernst, Reinhardt Ernst (2010) Vol. 169.

[143] Bienstein C Fröhlich A Basale Stimulation in der Pflege. Die Grundlagen. Bern: Hogrefe (2016).

[144] Aaltonen M Raitanen J Forma L Pulkki J Rissanen P Jylha M Burdensome transitions at the end of life among long-term care residents with dementia. J Am Med Dir Assoc (2014) 15(9):643–8. 10.1016/j.jamda.2014.04.018 24913211

[145] Bowling A Rowe G Adams S Sands P Samsi K Crane M Quality of life in dementia: a systematically conducted narrative review of dementia-specific measurement scales. Aging Ment Health (2015) 19(1):13–31. 10.1080/13607863.2014.915923 24881888

[146] Volicer L Simard J Palliative care and quality of life for people with dementia: medical and psychosocial interventions. Int Psychogeriatr (2015) 27(10):1623–34. 10.1017/S1041610214002713 25573531

[147] Volicer L van der Steen JT Outcome Measures for Dementia in the Advanced Stage and at the End of Life. Adv Geriatr (2014) 1–10. 10.1155/2014/346485

[148] Ettema TP Droes RM de Lange J Mellenbergh GJ Ribbe MW QUALIDEM: development and evaluation of a dementia specific quality of life instrument. Scalability, reliability and internal structure. Int J Geriatr Psychiatry (2007) 22(6):549–56. 10.1002/gps.1713 17152121

[149] Becker S Kruse A Schroder J Seidl U [The Heidelberg instrument for the assessment of quality of life in dementia (H.I.L.DE.)–dimensions of quality of life and methods of organization]. Z Gerontol Geriatr (2005) 38(2):108–21. 10.1007/s00391-005-0297-7 15868349

[150] NICE Dementia: assessment, management and support for people living with dementia and their carers. NICE guideline. National Institute for Health and Care Excellence (NICE) (2018). 30011160

[151] Palan Lopez R Mitchell SL Givens JL Preventing Burdensome Transitions of Nursing Home Residents with Advanced Dementia: It’s More than Advance Directives. J Palliat Med (2017) 20(11):1205–9. 10.1089/jpm.2017.0050 PMC5672615 28504894

[152] Sampson EL Lodwick R Rait G Candy B Low J King M Living With an Older Person Dying From Cancer, Lung Disease, or Dementia: Health Outcomes From a General Practice Cohort Study. J Pain Symptom Manage (2016) 51(5):839–48. 10.1016/j.jpainsymman.2015.12.319 26891605

